# Soft and Stretchable Polymeric Optical Waveguide-Based Sensors for Wearable and Biomedical Applications

**DOI:** 10.3390/s19173771

**Published:** 2019-08-30

**Authors:** Jingjing Guo, Changxi Yang, Qionghai Dai, Lingjie Kong

**Affiliations:** 1 State Key Laboratory of Precision Measurement Technology and Instruments, Department of Precision Instruments, Tsinghua University, Beijing 100084, China; 2 Department of Automation, Tsinghua University, Beijing 100084, China

**Keywords:** wearable and implantable devices, polymer optical waveguides, optical sensing and sensors

## Abstract

The past decades have witnessed the rapid development in soft, stretchable, and biocompatible devices for applications in biomedical monitoring, personal healthcare, and human–machine interfaces. In particular, the design of soft devices in optics has attracted tremendous interests attributed to their distinct advantages such as inherent electrical safety, high stability in long-term operation, potential to be miniaturized, and free of electromagnetic interferences. As the alternatives to conventional rigid optical waveguides, considerable efforts have been made to develop light-guiding devices by using various transparent and elastic polymers, which offer desired physiomechanical properties and enable wearable/implantable applications in optical sensing, diagnostics, and therapy. Here, we review recent progress in soft and stretchable optical waveguides and sensors, including advanced structural design, fabrication strategies, and functionalities. Furthermore, the potential applications of those optical devices for various wearable and biomedical applications are discussed. It is expected that the newly emerged soft and stretchable optical technologies will provide a safe and reliable alternative to next-generation, smart wearables and healthcare devices.

## 1. Introduction

With the increasing demand for real-time health monitoring and medical treatments, continuous attempts have been made to develop soft and stretchable sensor devices that could conformally interact with soft, curved surfaces and time-dynamic tissues of the human body [1,2]. Such devices have been designed with various functionalities for wearable/implantable monitoring of physiological (e.g., heart rate, body temperature) [3,4,5,6,7,8,9,10,11], mechanical (e.g., pressure, strain, torsion) [12,13,14,15,16,17,18,19,20,21], and biochemical (e.g., glucose, sweat, blood oxygen saturation) conditions [22,23,24,25,26] of the human body. Generally, stretchable sensors are realized by electronic strategies, depending on relative changes in their electrical characteristics (e.g., piezoelectricity, capacitance or resistance), which offer good sensitivity with simple readout [27]. However, these sensors normally require complex manufacturing processes and have poor biocompatibility due to the usage of electrically conductive components. Moreover, electronic devices are sensitive to electromagnetic interference (EMI) and suffer from electrical safety issues [28].

Optical waveguide-based platforms are promising alternatives to classical electronics for their distinct advantages such as EMI immunity, inherent electrical safety, and high stability in long-term wearing or implantation. Except for light transmission, waveguides can also be designed and functionalized for highly sensitive sensing (e.g., strain, temperature, and bioanalytes), through the modulation of light intensity, phase, wavelength, or polarization [28,29,30,31]. Despite the great promises, conventional optical waveguides are commonly made from solid and inorganic materials (e.g., silica glasses, silicon oxynitride), many of which unfortunately are not biocompatible [32]. Furthermore, waveguides made of these materials are highly stiff and rigid, resulting in a significant mechanical mismatch with the soft skins and tissues [33,34]. To address these limitations, new types of waveguides made from soft, stretchable, biocompatible, and even biodegradable materials have been intensively explored.

Here, we summarize recent developments in soft and stretchable optical waveguides, designed with optimal light-guiding capabilities and physiomechanical properties for wearing and/or implantation. In particular, we describe the various material designs, fabrication strategies, and functionalities of novel waveguides and highlight their potential for diverse applications in wearable and biomedical sensing.

## 2. Materials and Fabrication Strategies

As the alternatives to conventionally-used rigid materials such as glasses and plastics, there has been tremendous interest in developing waveguides from soft and elastic polymeric materials, offering biomechanical compatibility with the soft human skin and tissue [35]. To meet the demands of the light-guiding and biological applications, the chosen material and its optimization is critical. The most important properties of the polymeric materials for the waveguide design are their optical transparency and refractive index (RI), which dominate the light transmission length of the waveguides (or waveguide loss). The loss in the polymeric materials mainly arise from absorption of the constituent molecules and light scattering due to the structural imperfections. To optimize the material loss, approaches such as filtration, degassing and compositional adjustments (e.g., crosslinking density and molecular weight) may be adopted [32,34,36]. Besides transparency, the waveguide loss also depends on the RI of an optical material, which is vital for achieving effective light-guiding through the mechanism of so-called total internal reflection (TIR). To make TIR occur, the RI of the guiding materials should be larger than that of the surroundings, which could be a low-RI coating layer, for example, step-index optical fibers of core/cladding profile. In particular, for a single-layer waveguide, the surroundings might be air, water, or biological tissues, where TIR occurs at the waveguide boundaries.

Moreover, for implantable applications, high biocompatibility is demanded so as to avoid toxic or immunological responses of the hosting tissues. Conventional silica glasses possess superior optical properties for low-loss light-guiding, whereas they are not compatible with biological tissues, resulting in inflammation and immune reactions in long-term implantation [37]. Fabrication of optical waveguides from optically transparent polymeric materials with desirable biocompatibility and biodegradability are a promising alternative to address the above limitations. Table 1 summarizes some representative polymeric materials and the strategies for waveguide fabrication. In the following sections, the waveguides made from polymeric materials including hydrogels, biodegradable polymers, and elastomers will be further detailed and discussed.

### 2.1. Hydrogels

**Figure 1:**
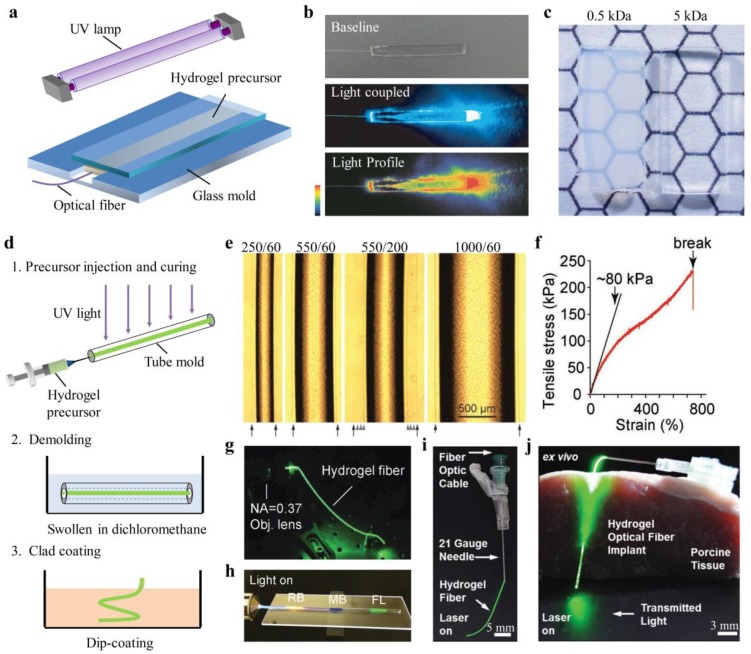
Hydrogel optical waveguides. (**a**) Fabrication method of a slab polyethylene glycol diacrylate (PEGDA) hydrogel waveguide [36]; (**b**) Light coupling to the slab hydrogel waveguide. Reproduced with permission [39]. Copyright 2013, Nature Publishing Group; (**c**) Photographs of the two hydrogel samples with molecular weight of 0.5 kDa and 5 kDA. Reproduced with permission [36]. Copyright 2013, Nature Publishing Group; (**d**) Fabrication steps of a step-index, core-cladding hydrogel optical fiber [32]; (**e**) Phase-contrast images of the core-cladding hydrogel fibers of different sizes. Reproduced with permission [32]. Copyright 2015, Wiley; (**f**) Stress-strain curve of a stretchable and tough hydrogel optical fiber. Reproduced with permission [39]. Copyright 2016, Wiley; (**g**) Light-guiding in a tough hydrogel optical fiber. Reproduced with permission [39]. Copyright 2016, Wiley; (**h**) Hydrogel optical fiber doped with multiple dye molecules for multiplexed strain sensing. RB, rhodamine B; MB, methylene blue; FL, fluorescein. Reproduced with permission [39]. Copyright 2016, Wiley; (**i**) Hydrogel optical fibers integrated with needles to create insertable devices. Reproduced with permission [40] Copyright 2017, Wiley; (**j**) Hydrogel optical fibers implanted in porcine tissue. Reproduced with permission [40]. Copyright 2017, Wiley.

Hydrogels are crosslinked, three-dimensional networks of hydrophilic polymers that can hold a large amount of water. In the past decades, hydrogels have been extensively investigated for a wide range of biomedical applications such as tissue engineering, wound dressings, and drug delivery, due to their soft and rubbery nature as well as high water content—similar to natural soft tissues [38]. Therefore, hydrogels with optimized optical and mechanical properties are favorable materials for light-guiding in tissues. Choi et al. fabricated slab and biocompatible optical waveguide from polyethylene glycol diacrylate (PEGDA) hydrogels by mold injection and ultraviolet (UV)-induced polymerization processes (Figure 1a,b) [36]. The optical, mechanical, and swelling properties of the PEGDA hydrogels could be controlled by adjusting the molecular weight, monomer concentrations, and waveguide structures for optimal light-guiding properties and functionalities. It was found that the hydrogels became more transparent and flexible as the molecular weight of PEGDA increased (Figure 1c). With a molecular weight of 10 kDa and a concentration of 10% w/v, PEGDA hydrogels showed notable transparency with an average loss as low as 0.17 dB/cm in the wavelength range of 450–550 nm. The hydrogel waveguide was integrated with fluorescent reporter cells and implanted in living mice for toxicity sensing [36].

Light-guiding in a single-layer waveguide is susceptible to the RI changes of the surrounding medium. For effective light confinement, step-index hydrogel optical fibers with core-cladding structures have been developed [32]. The fiber core was made from PEGDA hydrogel with a relatively large RI of ~1.45 through a molding process, while the cladding layer, made of calcium-alginate hydrogel (RI, ~1.34) was added to the core by dip coating (Figure 1d). Figure 1e shows the microscope images of the core-cladding hydrogel fibers with different sizes. The core diameter was determined by the inner diameter of the tube mold, and the cladding thickness could be controlled by the temperature of the dipping solution and the dipping times. The propagation loss of these hydrogel fibers was about 0.32 dB/cm in air and 0.49 dB in tissue. The feasibility of the hydrogel fibers for light-based therapies and sensing of blood oxygenation levels have also been tested in living mice [32].

Hydrogel optical waveguides are often brittle and fragile upon strain deformations due to the low mechanical strengths of common synthetic hydrogels. This characteristic hinders their applications in wearable or implantable sensing, where the body/tissue movement may induce structural damage to the waveguides. To achieve high deformability and robustness, hybrid ionic/covalent hydrogels with high stretchability and toughness were employed for the fabrication of optical fibers. Stretchable and tough hydrogel optical fibers with overall step-index profile have been made from hybrid alginate-polyacrylamide hydrogels by molding and dip-coating (Figure 1f,g) [39]. Upon stretching, the ionic chains of Ca^2+^-alginate breaks, providing an energy dissipation, while the covalently crosslinked, long-chain polyacrylamide (PAAm) gives high deformability and maintains the mechanical integrity of the hydrogels. As a result, the tough hydrogel optical fiber was endurable to a large tensile strain of up to 700% (Figure 1f). To avoid the delamination of the core and cladding under large deformations, the cladding was covalently bonded to the core by using EDC-NHS chemistry. Harnessing the unique mechanical properties, the tough hydrogel fiber was doped with dye molecules for large strain sensing with multiplexing capabilities based on absorption spectroscopy (Figure 1h) [39]. Another work reported an elastic and implantable hydrogel optical fiber using a copolymer of PEGDA–PAAm as the core and alginate hydrogel as the cladding [40]. Such a hydrogel fiber was functionalized with phenylboronic acid to monitor glucose concentrations from the intensity changes of light passing through the fiber. Integrated with needles, the hydrogel fiber could be used as a flexible and insertable device for implantation in tissues (Figure 1i,j).

### 2.2. Biodegradable Polymers

Naturally derived polymers with a high degree of biocompatibility and biodegradability have been widely used in medical and clinical applications, such as for controlled drug release, cell encapsulation, and regenerating and repairing damaged tissue [41]. A variety of natural polymeric materials, such as silk [42,43,44,45,46], agarose [47,48], and cellulose [49] have been demonstrated for waveguide fabrication. Waveguides made from biodegradable polymers could degrade and be absorbed by the human body, so they could remain within the body after use, eliminating the need for surgical removal.

**Figure 2:**
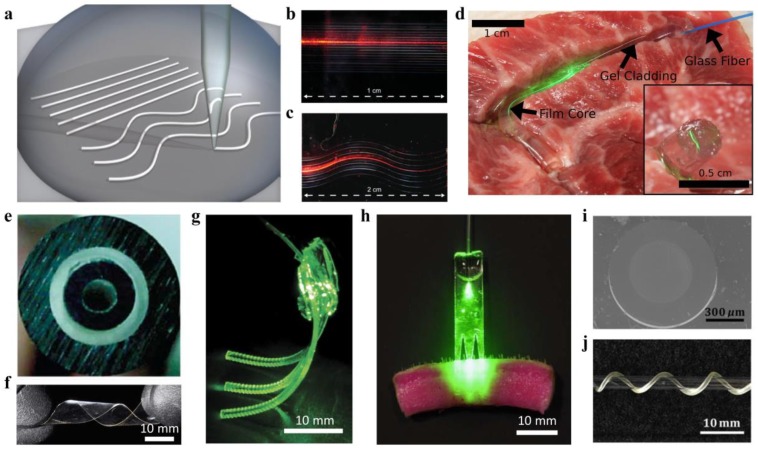
Biodegradable optical waveguides. (**a**) Schematic of direct printing of silk waveguides on borosilicate glass slides. Reproduced with permission [42]. Copyright 2009, Wiley; (**b,c**) Images of straight (**b**) and wavy (**c**) silk waveguides when a He-Ne laser source was launched. Reproduced with permission [42]. Copyright 2009, Wiley; (**d**) Light-guiding of silk waveguides in tissue. Reproduced with permission [44]. Copyright 2015, Optical Society of America; (**e**) Cross-section image of a biodegradable double-core optical fiber made from cellulose. Reproduced with permission [49]. Copyright 2007, Optical Society of America; (**f**) Mechanical flexibility of a polylactic acid (PLA) film. Reproduced with permission [51]. Copyright 2016, Nature Publishing Group; (**g,h**) Bioabsorbable PLA waveguide array for photochemical tissue bonding (PTB) treatment of porcine skin incision. Reproduced with permission [51]. Copyright 2016, Nature Publishing Group; (**i**) Cross-section image of a citrate-based optical fiber with poly(octamethylene maleate citrate) (POMC) core and poly(octamethylene citrate) (POC) cladding. Reproduced with permission [55]. Copyright 2017, Elsevier; (**j**) Photograph of a twisted citrate-based optical fiber around a glass tube, showing mechanical flexibility. Reproduced with permission [55]. Copyright 2017, Elsevier.

Among all, silk fibers, produced by worms and spiders, are one of the most attractive natural polymers for bio-optical designs due to their high degree of biodegradability and mechanical toughness [50]. Parker et al. developed a microscale silk optical waveguide with a high RI of ~1.54 through direct printing of an aqueous silk fibroin ink on borosilicate glass slides (Figure 2a) [42]. The silk waveguides printed in straight and wavy structures could effectively guide light in air with an optical loss of 0.25 dB/cm and 0.81 dB/cm, respectively, at 633 nm (Figure 2b,c). Spin-coating techniques have also been used to deposit silk planar waveguides on borosilicate glasses and Si/SiO_2_ substrates, which showed optical losses of 0.22 dB/cm at 632.8 nm [43]. Despite the low loss, these substrate-integrated waveguides were not free-standing, which poses limitations for their use in vivo. To overcome the shortcoming, step-index, core-cladding optical waveguides made of silk fibroin were created by mold injection [44]. The core of the waveguide was a silk film with a high RI of 1.54; while the cladding was a silk hydrogel, having a low RI of ~1.34. The high RI contrast enabled strong guidance of light in tissue with an optical loss of ~2 dB/cm (Figure 2d). Besides light-guiding, multiple functionalities could be integrated into polymer waveguides with specialized structural designs. Dupuis et al. developed a cellulose-based biodegradable optical fiber with a porous double-core structure, integrating multiple functionalities of light-guiding, microfluidics, and drug delivery [49]. The optical fiber was formed by thermally drawing two cellulose butyrate tubes with cellulose powders in between (Figure 2e). The transmission loss of the cellulose optical fiber was ~1 dB/cm. When filled with water, the fiber microstructure was fully dissolved after one day.

Additionally, some synthetic degradable polymers, such as polylactic acid (PLA), poly(lactic-co-glycolic) (PLGA), polyglycolic acid (PGA), and citrate-based polymers have also been exploited to develop biodegradable waveguides [51,52,53,54,55]. For example, Nizamoglu et al. proposed a PLA-based bioabsorbable planar waveguide through melt pressing, and demonstrated its application in photochemical tissue bonding (PTB) treatment of porcine skin incision (Figure 2f–h) [51]. Shan et al. reported a biodegradable step-index optical fiber by mold injection, where the core material was poly(octamethylene maleate citrate) (POMC) and cladding was poly(octamethylene citrate) (POC) (Figure 2i,j) [55]. With a low guiding loss of 0.4 dB/cm, the biodegradable optical fiber was utilized for image transmission, deep-tissue light delivery, and fluorescence sensing in vivo.

### 2.3. Elastomers

**Figure 3:**
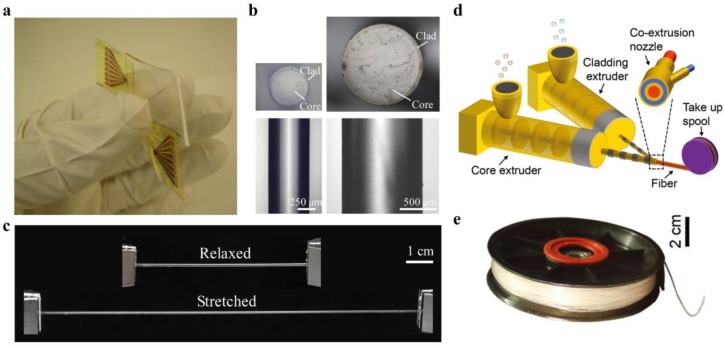
Elastomer optical waveguides. (**a**) Bendable and stretchable polydimethylsiloxane (PDMS) waveguide array for optical linking between laser sources and photodiodes. Reproduced with permission [57]. Copyright 2015, Optical Society of America; (**b**) Cross-section images of step-index, core-cladding PDMS optical fibers. Reproduced with permission [64]. Copyright 2019, Wiley; (**c**) High stretchability of the PDMS optical fiber. Reproduced with permission [64]. Copyright 2019, Wiley; (**d**) Coextrusion fabrication of step-index polymer optical fibers from thermoplastic elastomers. Reproduced with permission [68]. Copyright 2018, Wiley; (**e**) The 200 m fiber fabricated in one hour through the coextrusion process. Reproduced with permission [68]. Copyright 2018, Wiley.

Waveguides with high flexibility and stretchability are demanded to develop optical wearables for health monitoring. Although hydrogel materials, such as Alginate-PAAm, could offer large deformability, they are not suitable to work in dry conditions due to their large water content. To address this problem, many researchers developed waveguides from organic elastomers. Polydimethylsiloxane (PDMS) is a silicone-based organic elastomer, particularly known for its high elasticity, chemical inertness, and thermal stability [56,57,58]. Moreover, PDMS possesses high transparency over a wide spectral range, making it an excellent optical material for light guiding [57,58,59,60,61,62,63,64,65,66,67]. Bendable and stretchable PDMS planar waveguide array has been developed based on the molding and replication technique [57]. Integrated with sources and detectors, the waveguide array served as a flexible optical link that is endurable to mechanical deformations (Figure 3a). Prajzler et al. developed a core-cladding rectangular waveguide by using polydimethyl-diphenylsiloxane (PDMDPS) as the core and PDMS elastomers as the cladding. The waveguide had optical losses below 0.35 dB cm^−1^ at 532, 650, 850, and 1310 nm [60]. The RI of PDMS (Sylgard 184) can be changed by altering the curing temperature, density, or mixing ratio of the base and curing agent. Odeh et al. demonstrated a gradient-index (GRIN) optofluidic planar waveguide by applying a gradient temperature during the thermal curing of the PDMS [62]. The GRIN profile confined light around a microfluidic channel with a propagation loss of 1.47 dB/cm, promising for evanescent sensing applications. Cai et al. proposed a step-index, all-PDMS planar waveguide by spin coating, where the core and cladding were made by PDMS at different mixing ratios of base and curing agent for the different RIs [63]. The RI of the PDMS core (base/curing agent = 5:1) and cladding (base/curing agent=20:1) were 1.424 and 1.417, respectively. This RI contrast enabled effective light confinement with a propagation loss of 1.1 dB/cm. With similar RI settings, a highly stretchable, step-index PDMS optical fiber has been developed through mold injection and spin-coating (Figure 3b,c) [64]. Tensile tests were performed to characterize mechanical properties of the PDMS fiber (diameter, ~1 mm), where the fiber showed a low Young’s modulus of ~1.28 MPa and a stretchability up to 134%, offering skin-like mechanical compliance. The potentials of the stretchable PDMS fiber for wearable health monitoring have also been demonstrated [64]. In addition, researchers have also achieved single-mode rib waveguides with microscale core size (<8 µm) by soft lithography techniques, where pure PDMS (RI, 1.412) and hexane-modified PDMS (RI, 1.411) were used for the core and cladding, respectively [65]. The propagation loss of the single-mode rib waveguide was measured to be 0.48 dB/cm at 635 nm.

Soft polymer optical fibers are generally fabricated through a molding process, followed by curing and demolding, which are difficult to achieve for fibers at a large length scale (>100 m). To overcome this limitation, recent attempts have been made to produce optical fibers from thermoplastic elastomers due to their melt-processability [68,69,70]. Leber et al. reported scalable fabrications of highly stretchable, core-cladding optical fibers through a one-step coextrusion process (Figure 3d) [68]. The core and cladding were made of polystyrene-based polymer Star Clear 1044 (RI, 1.52) and fluorinated polymer Daikin T-530 (RI, 1.36), respectively. Following this approach, they fabricated fibers with 200 m length in one hour of continuous coextrusion (Figure 3e). The core-cladding fibers showed excellent mechanical flexibility and stretchability that could reversibly sustain large tensile strains of up to 300%. The propagation loss of these fibers was 0.8–1.2 dB/cm at visible wavelengths.

## 3. Applications of Polymeric Optical Waveguide-Based Sensors

### 3.1. Implantable Waveguides for Biomedical Sensing

Biocompatible polymeric optical waveguides with soft and flexible mechanical characteristics have shown great potentials in a broad range of biomedical applications in sensing, diagnosis, and phototherapy [71,72]. In this section, we focus on their applications in biomedical sensing, where waveguide sensors, serving as implantable and injectable devices, can be used for continuous and long-term monitoring of biological information inside the body and tissue. 

**Figure 4:**
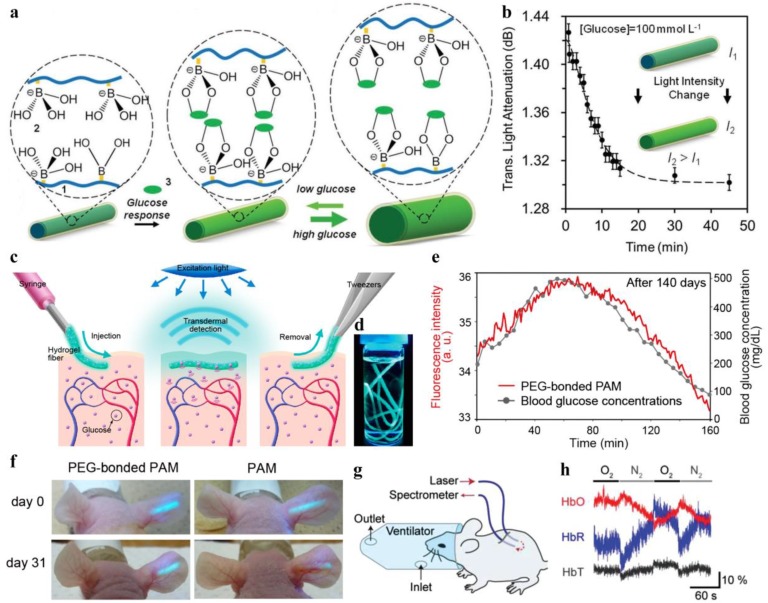
(**a**) Schematic diagram of a hydrogel optical fiber functionalized with 3-APBA for glucose monitoring. Reproduced with permission [40]. Copyright 2017, Wiley; (**b**) Intensity changes of the transmitted light through the glucose-sensitive fiber over time at a glucose concentration of 100 mmol L^−1^. Reproduced with permission [40]. Copyright 2017, Wiley; (**c**) Injectable fiber incorporated with fluorescent glucose-responsive monomer for long-term monitoring of blood glucose concentration in vivo. Reproduced with permission [73]. Copyright 2011, National Academy of Sciences; (**d**) Photograph of fluorescent fibers in a glass vial. Reproduced with permission [73]. Copyright 2011, National Academy of Sciences; (**e**,**f**) Implantation of fluorescent fibers in the ear of mice for glucose monitoring. Reproduced with permission [73]. Copyright 2011, National Academy of Sciences; (**g**,**h**) Two core-cladding hydrogel optical fibers implanted in mouse subcutaneous tissue to measure oxygenated and deoxygenated hemoglobin concentrations. Reproduced with permission [32]. Copyright 2015, Wiley.

Continuous monitoring of glucose levels in the blood is of great significance for management and treatment of diabetes mellitus in clinics. Conventional plastic optical fibers modified with glucose sensing receptors have been previously exploited for continuous intravascular glucose monitoring in clinics [37]. However, due to their poor biocompatibility, long-term implantation of these fibers could induce severe inflammation and immune reactions. Recently, biocompatible, step-index hydrogel optical fibers functionalized with phenylboronic acid (PBA) have been demonstrated for glucose sensing [40]. Figure 4a shows the schematic diagram of a glucose-sensitive, biocompatible hydrogel optical fiber. The hydrogel fiber was comprised of a core of PEGDA-PAAm copolymer and a cladding of alginate hydrogel, in which the core was covalently bonded with 3-(acrylamido)phenylboronic acid (3-APBA) molecules. The porous matrices of the hydrogel enabled glucose molecules to diffuse into the fiber and complex with 3-APBA, resulting in volume swelling of the fiber. Quantitative and real-time monitoring of glucose was achieved by measuring the intensity changes of the transmitted light, induced by fiber swelling (Figure 4b). Heo et al. developed an injectable hydrogel fiber, which was incorporated with fluorescent glucose-responsive monomers for long-term glucose monitoring [73]. The hydrogel fibers were made by PAAm hydrogel coated with polyethylene glycol (PEG) for enhanced biocompatibility. Due to their small diameters, the hydrogel fibers could be easily injected into the body through a syringe needle (Figure 4c,d). They implanted the fluorescent fibers in the ear of mice and achieved continuous monitoring of blood glucose concentration for up to 140 days (Figure 4e,f). 

**Figure 5:**
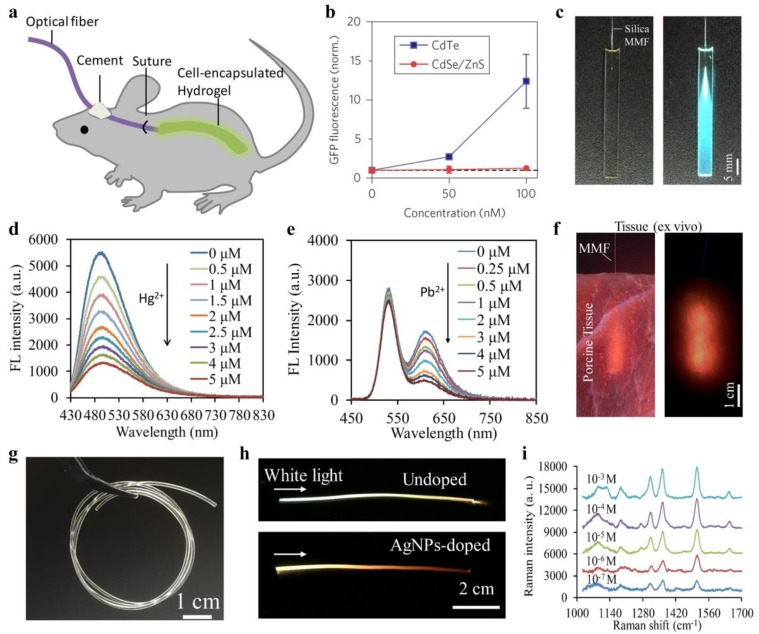
(**a**,**b**) Cell-encapsulated hydrogel waveguide implanted in living mice for toxicity sensing of cadmium-based QDs. Reproduced with permission [36]. Copyright 2016, Nature Publishing Group; (**c,d**) CDs-doped fluorescent waveguide for metal-ion sensing. Reproduced with permission [75]. Copyright 2017, Nature Publishing Group; (**e**) Waveguides loaded with two types of QDs at different emission bands for ratiometric sensing of metal ions. Reproduced with permission [76]. Copyright 2018, American Chemical Society; (**f**) Demonstration of the fluorescent waveguide for ratiometric sensing in tissues. Reproduced with permission [76]. Copyright 2018, American Chemical Society; (**g**) Side view of a PEGDA hydrogel optical fiber. Reproduced with permission [34]. Copyright 2018, Optical Society of America; (**h**) Photographs of hydrogel fibers before and after silver nanoparticles (AgNPs) doping upon white light illumination. Reproduced with permission [34]. Copyright 2018, Optical Society of America; (**i**) Surface-enhanced Raman scattering (SERS) spectra of R6G molecules at various concentrations measured by AgNPs-doped hydrogel optical fiber. Reproduced with permission [34]. Copyright 2018, Optical Society of America.

Besides glucose monitoring, biocompatible optical fibers have also been investigated for blood oxygenation sensing by absorbance spectroscopy [32]. Two core-cladding hydrogel optical fibers with their tips separated by 5 mm were implanted in mouse subcutaneous tissue, where one of the hydrogel fibers was used for light delivery into the tissue and the other one was for light collection (Figure 4g). The relative oxy- and deoxyhemoglobin concentrations in blood were obtained from the changes of light intensity based on Beer–Lambert law (Figure 4h). Hydrogels are excellent cell scaffolds due to their high porosity and biomimetic properties. Choi et al. encapsulated sensing cells into a slab PEGDA hydrogel waveguide and applied it for toxicity sensing of cadmium-based quantum dots (QDs) in vivo [36]. The cell-containing hydrogel waveguide was implanted in living mice, where toxic QDs (CdTe; CdSe/ZnS) could be detected from the fluorescence signals of the sensing cells (Figure 5a,b). Forster et al. presented an implantable hydrogel waveguide with integrated fluorescent proteins for calcium detection, where the calcium ions bond with the immobilized proteins, resulting in fluorescence changes due to the fluorescence resonance energy transfer effect [74]. Also, implantable optical waveguides coupled with fluorescence sensing have been exploited as optical neural interfaces for studying neural activity in vivo [33,52].

Furthermore, functional nanoparticles such as QDs and silver/gold nanoparticles could be incorporated into biocompatible waveguides through chemical bonding or physical entrapping, which endows the waveguides with new sensing functionalities. In recent work, researchers developed a carbon dots (CDs)-doped slab hydrogel waveguide for Hg^2+^ sensing (Figure 5c) [75]. The hydrogel waveguide served as a light-guiding platform, allowing laser excitation of the CDs and emission collection, while small Hg^2+^ ions could penetrate into the porous hydrogel matrices and quench the CDs. Coupled with standard silica optical fibers, they achieved selective and sensitive detection of Hg^2+^ from the fluorescence quenching, attributed to the nonradiative electron transfer of the incorporated CDs (Figure 5d). Fluorescence intensity-based sensing is susceptible to interferences such as fluctuations of the excitation laser power, changes of the surrounding environments, and instrumental efficiency. To minimize the analyte-independent effects, hydrogel waveguides doped with two types of QDs at different emission bands were demonstrated for ratiometric sensing of metal ions (Figure 5e) [76]. The ratiometric configuration offered a built-in calibration that makes the waveguide sensor robust to environmental perturbations. The feasibility of such QDs-doped waveguide for optical sensing in tissues has been tested (Figure 5f). In addition, hydrogel optical fibers have also been doped with silver nanoparticles (AgNPs) for in-su surface-enhanced Raman scattering (SERS) sensing (Figure 5g,h) [34]. The AgNPs-doped hydrogel optical fibers, having a Young’s modulus comparable to soft biological tissues, were demonstrated for detection and discrimination of bioanalytes from the SERS spectra (Figure 5i).

### 3.2. Wearable Physiological Monitoring of the Human Body

**Figure 6:**
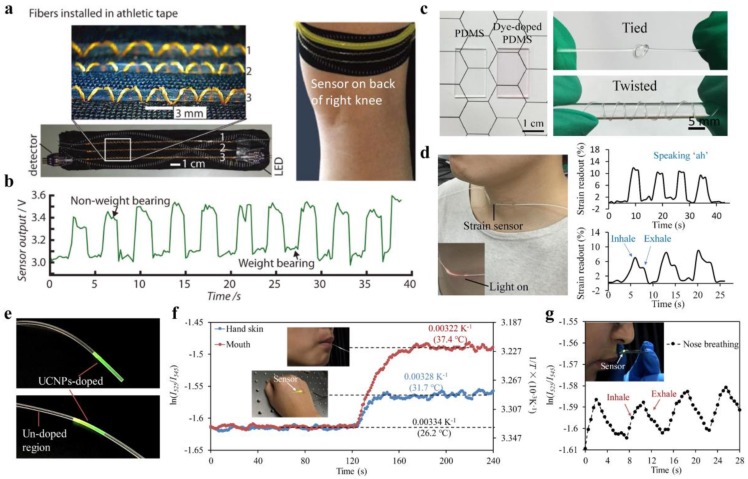
(**a**,**b**) Stretchable fiber-based strain sensor installed on an athletic tape to monitor weight-bearing activities. Reproduced with permission [70]. Copyright 2017, Wiley; (**c**,**d**) Dye-doped PDMS fiber developed for wearable strain sensing during speaking and deep breathing. Reproduced with permission [83]. Copyright 2017, Optical Society of America; (**e**) Photographs of upconversion nanoparticles (UCNPs)-doped fibers under 980 nm excitation. Reproduced with permission [64]. Copyright 2019, Wiley; (**f**,**g**) Demonstration of the UCNPs-doped fibers in real-time temperature monitoring of surface skin and interior mouth (**f**), as well as thermal signals generated by nose breathing (**g**). Reproduced with permission [64]. Copyright 2019, Wiley.

For wearable and skin-comfortable sensing, sensors with high flexibility and deformability are demanded to ensure high mechanical compliance with the soft and curvilinear surfaces of the human body. In the past decades, a variety of stretchable electronic materials and structures have been exploited to achieve wearable sensing devices for monitoring physiological signals, such as temperature, strain, and pressure [77]. However, these sensors are often in need of complex production processes and are susceptible to EMI and electrical safety issues (e.g., current leakage). Alternatively, fiber-optic sensors have been investigated extensively for wearable health monitoring due to their attractive advantages including EMI immunity, electrical safety, and miniaturized size [78,79]. For example, an intensity-modulated microbend fiber-optic sensor has been developed for noninvasive monitoring of respiratory in strong electromagnetic interference environments during magnetic resonance imaging (MRI) [80]. Fiber Bragg grating sensors have been integrated into functional textiles for wearable monitoring of body temperature [81]. Nevertheless, conventional fiber-optic sensors utilizing silica optical fibers are far too rigid compared with human skins, which restricts the natural movements of the wearer. Moreover, the high rigidity of silica fibers poses a limit for many applications such as human motion detection, where large deformation needs to be accommodated by the sensor. The implementation of wearable sensors by using stretchable optical waveguides offer a promising approach to overcome the limitations above. To et al. reported a stretchable, gold-coated PDMS optical waveguide for wearable detection of human motions [82]. A light-emitting diode and a photodiode were embedded at each end of the waveguide, serving as the light source and detector, respectively. When deformed, the waveguide showed microcracks within the reflective gold layer, enabling measurements of pressure, strain, and curvature from the changes of light transmission. Harnett et al. demonstrated a stretchable and strain-sensitive optical fiber, formed with a polyurethane core and a silicone cladding [70]. The stretchable optical fiber, capable of sustaining >100% strains, was assembled into textiles using a sewing machine (Figure 6a). The attaching thread caused microbends in the fiber upon stretching, resulting in monotonic transmission changes against strain. The sewn fibers were installed on a piece of athletic tape for wearable monitoring of strains generated by weight-bearing activities, which could be used in sports monitoring and functional fitness tests (Figure 6a,b). Detection of strains from the changes of light intensities induced by waveguide deformations suffers from a nonlinear response behavior [68,70,82]. Recently, a stretchable optical strain sensor with high linearity has been developed by using dye-doped PDMS optical fiber (Figure 6c) [83]. The basic principle of such a strain sensor was based on the absorption of dye molecules following Beer–Lambert law, in which the fiber loss increased linearly with the fiber length upon stretching. The potentials of these optical strain sensors in wearable monitoring of various human activities, such as speaking and deep breathing, have been demonstrated (Figure 6d). 

In addition to mechanical sensing, stretchable polymeric optical waveguides have also been investigated for wearable measurements of body temperature [64]. Body temperature is one of the most significant physiological signals that effectively indicates health states. For continuous and long-term temperature monitoring, the readout of wearable temperature sensors should be robust to body movements and independent of mechanical deformation. A novel stretchable optical temperature sensor, capable of maintaining its sensing performance under large strain deformations (up to 80%), has been developed by using upconversion nanoparticles (UCNPs)-incorporated PDMS optical fibers (Figure 6e) [64]. The PDMS optical fiber, comprised of a step-index core-cladding structure, enabled effective excitation of the luminescent UCNPs. A linear sensing range of 20–70 °C was achieved by ratiometric measurements of the dual-wavelength UCNPs emissions upon near-infrared (NIR) excitation. By virtue of the ratiometric readout, the temperature sensor was self-calibrated and its sensing performance was independent of strain deformations. To demonstrate its wearable potentials, the temperature sensor was used to monitor both the interior and surface temperatures of the human body (Figure 6f,g).

**Figure 7:**
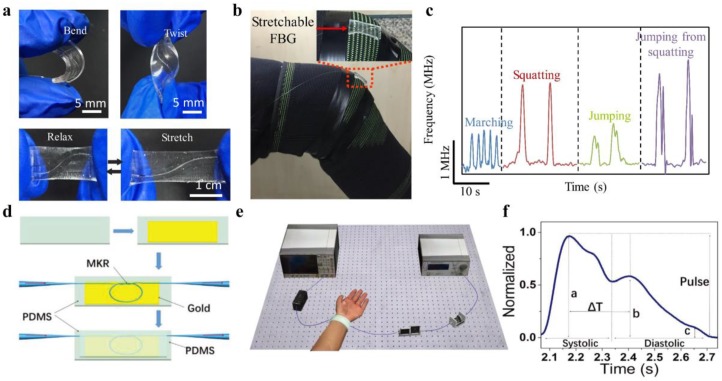
(**a**) A stretchable fiber-optic strain sensor, fabricated by incorporating sinuous-shaped fiber-Bragg-grating (FBG) in a slab PDMS substrate, which could be bent, twisted, and stretched like skin. Reproduced with permission [84]. Copyright 2019, Wiley; (**b**,**c**) Stretchable FBG sensor installed on an elastic kneepad to monitor various knee-related sports activities. Reproduced with permission [84]. Copyright 2019, Wiley; (**d**) Schematic illustration of a flexible optical strain sensor based on a microfiber resonator situated on a smooth gold film. Reproduced with permission [85]. Copyright 2018, Wiley; (**e**,**f**) Wearable measurement of wrist pulses with the microfiber sensor. Reproduced with permission [85]. Copyright 2018, Wiley.

Furthermore, there have also been efforts to enhance flexibility and elasticity of conventional silica fiber-based sensors through material coating and structural designs for wearable applications. A stretchable fiber-optic strain sensor made up of a sinuous-shaped fiber-Bragg-grating (FBG) embedded in a slab PDMS substrate has been fabricated by using 3D-printed mold [84]. The sinuous-shaped structure of FBG enabled sensing of various deformations including tension, bending, and torsion from the Bragg wavelength shift (Figure 7a). Multiplexing capabilities of the FBG-based sensors have been explored by using a free-running fiber laser based on dual-comb spectroscopy. The stretchable sensor was attached to an elastic kneepad and applied for sports activities monitoring (Figure 7b,c). Li et al. reported a flexible optical microfiber sensor for strain and pressure detections by embedding a hybrid plasmonic microfiber resonator in a PDMS membrane (Figure 7d) [85]. The operating principle of the microfiber sensor for strain and pressure sensing was based on the shift of the resonating wavelength, ascribed to the changes of light polarization and photoelasticity of PDMS under mechanical deformations. The flexible microfiber resonator was worn on the human wrist to detect artery pulse, which is an important indicator of heartbeat and arterial blood pressure (Figure 7e,f). These results showed great promise for the optical devices in wearable heath monitoring and medicines.

### 3.3. Human–Machine Interfacing and Robotics

**Figure 8:**
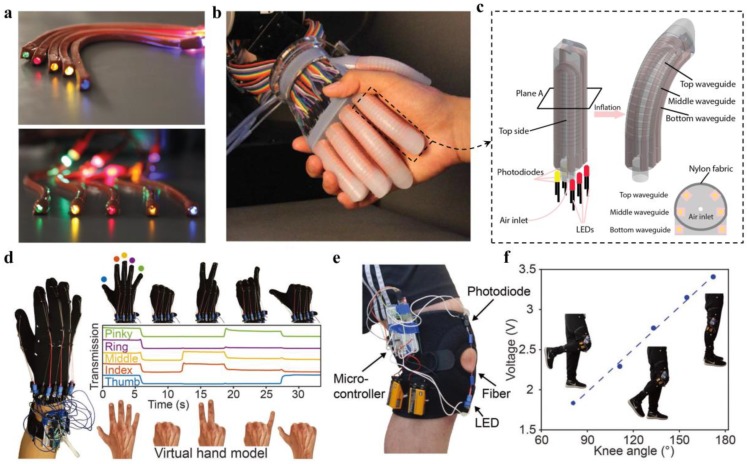
(**a**) Strain-sensitive optical waveguide comprised of a core of polyurethane rubber and the cladding of silicone composite. Reproduced with permission [86]. Copyright 2016, American Association for the Advancement of Science; (**b**) Shaking with a prosthetic hand, integrated with stretchable optical waveguides for strain sensing. Reproduced with permission [86]. Copyright 2016, American Association for the Advancement of Science; (**c**) Schematic illustration of the prosthetic innervated finger integrated with sensory waveguides. Reproduced with permission [86]. Copyright 2016, American Association for the Advancement of Science; (**d**) Smart glove. Stretchable core-cladding optical fibers made from thermoplastic elastomers were installed on each finger of a glove to track the finger movements, enabling real-time control of a virtual hand model. Reproduced with permission [68]. Copyright 2018, Wiley; (**e**,**f**) Stretchable sensory fiber integrated with an LED and a photodiode to track the knee angle. Reproduced with permission [68]. Copyright 2018, Wiley.

Interactive human–machine interface (HMI) of a robotic system enables information transfer between human and machine for remote manipulation and feedback collection. To achieve intelligent HMI, wearable sensory systems are essentially in need. Zhao et al. developed a soft prosthetic hand with haptic sensation by integrating stretchable and strain-sensing optical waveguides for curvature, elongation, and force measurements (Figure 8a–c) [86]. The sensory waveguides, consisting of a core of polyurethane rubber (RI, 1.461) and a cladding of silicone composite (RI, 1.389), were fabricated by the soft lithography processes. For optical interrogation and detection, the waveguides were designed with two holes to hold the light-emitting diode and photodiode, respectively (Figure 8c). As demonstrations, they showed the capabilities of the robotic hand in dexterous manipulation and haptic sensing to mimic the functions of a real hand [86]. 

Smart gloves for quantifying hand movements have also been developed by the use of stretchable optical fibers [68]. The stretchable fibers, capable of holding strains up to 300%, were made from thermoplastic elastomers by a continuous coextrusion process. Deformations of the fiber resulted in reversible and detectable changes in its light transmission, enabling deformation sensing from the transmitted light intensities. The fibers were installed on the finger joints of a glove, where each finger motion could be measured and distinguished from the corresponding sensor output, allowing identification of hand gestures. Figure 8d shows the real-time control of a virtual hand model by wearing the smart glove, indicating great potentials of being used as an input gear for virtual reality and interactive games. Additionally, the sensory fibers could also be used to track the evolution of the knee angle during walking or running, enabling applications in the assessment of athletic performance, knee rehabilitation, and assisting human/robot walking (Figure 8e,f) [68]. The waveguide-based stretchable sensors provide a safe, reliable, and innovative optical approach towards wearable sensing, which should benefit and push forward the fields of human–machine interfacing and robotics.

**Table 1:** Representative polymeric materials and strategies for waveguide fabrication.

Category	Structure	Material	Strategy	Reference
Hydrogel waveguides	Core-cladding	PEGDA/Alginate	Molding; Dip-coating	[32]
		PEGDA-PAAm/Alginate		[39]
		Alginate-PAAm		[40]
	Single-layer	PEGDA	Molding	[34,36,75]
Biodegradable waveguides	Core-cladding	Silk	Molding	[44]
		POMC/POC	Molding	[55]
		Agarose/Gelatin	Spin-coating	[47]
		Agarose	Lithography	[48]
	Porous double-core	Cellulose	Thermal drawing	[49]
	Single-layer	Silk	Printing; Spin-coating	[42,45,46]
		PLA	Melting and laser cutting; Thermal drawing	[51,52]
		PLGA		[51]
Elastomer waveguides	Core-cladding	PDMS	Spin-coating; Mold injection; Lithography;	[63,64,65,66]
		Star Clear 1044/Daikin T-530	Coextrusion after melting	[68]
		Polyurethane/PDMS	Drawing	[70]
	Single-layer	PDMS	Mold injection; Printing; Drawing	[57,61,83]
Other polymeric waveguides	Core-cladding	Siloxane, Epoxy	Spin-coating; Lithography	[87,88,89,90]
		Benzocyclobutene	Spin-coating	[91]
		Butenylvinyleth	Laser writing	[92]

## 4. Conclusions and Outlook

We have reviewed recent progress on soft and stretchable polymeric optical waveguides for wearable and biomedical sensing applications. Optimization, functional modification, and fabrication strategies of polymeric materials including hydrogels, biodegradable polymers, and elastomers for various waveguide designs have been highlighted. Compared to conventional rigid silica or plastic optical waveguides, the developments of waveguide devices from optically transparent polymeric materials with soft, stretchable, and biocompatible features provides desired mechanical, chemical, and biological properties for long-term wearing and implantation. 

Hydrogels are among the most intriguing polymeric materials for light-guiding and optical sensing in tissues. The large water content and porous structures of hydrogels enable encapsulating living cells and functional molecules for innovative biosensing applications such as continuous glucose monitoring, metal-ion detection, and in vivo toxicity sensing. Besides, there have also been efforts in developing optical waveguides that are biodegradable. Naturally-derived polymers such as silk, agarose, and cellulose, are great choices for the design of biodegradable waveguides. In addition to their high biocompatibility, these biodegradable devices as implants could be left within the body after use, as they would gradually degrade and eventually be absorbed in the body. Moreover, polymeric optical waveguides with high deformability have been exploited as wearables over the body by using stretchable elastomers such as PDMS and polyurethane. The elastomer-based optical waveguides possessed skinlike mechanical compliance and could conformally interact with the soft and curvilinear surfaces of human body. With novel structural designs and principles, stretchable optical waveguides have been investigated for wearable sensing of strain, pressure, and temperature, which could find widespread applications, such as assessment of sports performances, motion capturing for virtual reality and interactive games, and remote robot control. 

Despite the great promises described above, there still remain many challenges for the implementation of polymeric waveguide-based sensors in practical applications. The implantable sensing waveguides are envisioned to be used in clinics from point-of-care diagnostics to therapeutic treatments. For further developments for clinical utility, the propagation loss of the polymer waveguides needs to be further decreased to ensure high signal-to-noise ratio and deeper implantation. The current waveguides generally possessed an optical loss of several dB/cm, which are five orders of magnitude higher than that of conventional silica fibers. The intrinsic loss of the waveguides can be minimized through optimization and adjustments of the material constituent; while the scattering loss, caused by surface roughness and structural defects, can be decreased by improving the fabrication strategies. Sensing functionalities of the waveguide can be further expanded by the exploration of novel structures, materials, and principles to provide comprehensive information inside the body for diagnostics and health tracking. To be used as wearables over the body, the optical sensing systems are required to be lightweight and have low-power-consumption. Instead of using bulky light sources and spectrometers, miniaturized light-emitting diode and photodiodes can be great choices. Wearable sensors with multiple sensory functions to mimic the human somatosensory system are crucial for developing artificial intelligent products. Although the wearable waveguide-based sensors have been previously developed for the sensation of various physiological signals, multiplexed sensing with a single waveguide device remains challenging. One promising approach is to integrate the polymeric waveguide with diverse sensing schemes (such as using fluorophores, plasmonic nanostructures, and gratings), where intensity/wavelength/polarization multiplexing technique may be adopted for the signal decoupling. These new classes of polymeric waveguides are expected to offer versatile optical platforms for advanced sensing in biomedicine, health monitoring, and human–machine interfaces.
